# Highly virulent avian brood-parasitic species show elevated embryonic metabolic rates at specific incubation stages compared to less virulent and non-parasitic species

**DOI:** 10.1098/rsbl.2024.0411

**Published:** 2024-09-25

**Authors:** Stephanie C. McClelland, Jess Lund, Tanmay Dixit, Silky Hamama, Luke A. McClean, Claire N. Spottiswoode, Craig R. White, Matthew I. M. Louder, Mark E. Hauber, Marcel Honza, Steven J. Portugal

**Affiliations:** ^1^ Department of Biological Sciences, School of Life and Environmental Sciences, Royal Holloway University of London, Egham, Surrey TW20 0EX, UK; ^2^ Department of Zoology, University of Cambridge, Downing Street, Cambridge CB2 3EJ, UK; ^3^ FitzPatrick Institute of African Ornithology, DST-NRF Centre of Excellence, University of Cape Town, Rondebosch, Cape Town 7701, South Africa; ^4^ c/o Musumanene Farm, Choma, Zambia; ^5^ Auckland University of Technology, Auckland 1010, New Zealand; ^6^ School of Biological Sciences, Monash University, Clayton, Victoria 3800, Australia; ^7^ Advanced Science Research Center and Programs in Biology and Psychology, Graduate Center of the City University of New York, New York, NY 10031, USA; ^8^ American Museum of Natural History, New York, NY 10024, USA; ^9^ The Czech Academy of Sciences, Institute of Vertebrate Biology, Květná 8, Brno 603 65, Czech Republic; ^10^ The Natural History Museum, Tring, Herts HP23 6AP, UK; ^11^ Department of Biology, The University of Oxford, Oxford OX1 3SZ, UK

**Keywords:** brood parasitism, embryo metabolism, cowbirds, cuckoos, honeyguides, respirometry

## Abstract

As the avian embryo grows and develops within the egg, its metabolic rate gradually increases. Obligate avian brood-parasitic birds lay their eggs in the nests of other species to avoid the costs of parental care, and all but one of these brood-parasitic species are altricial at hatching. Yet the chicks of some altricial brood-parasitic species perform the physically demanding task of evicting, stabbing or otherwise killing host progeny within days of hatching. This implies a need for high metabolic rates in the embryo, just as precocial species require. Using flow-through respirometry *in situ*, we investigated embryonic metabolic rates in diverse avian brood parasite lineages which either kill host offspring (high virulence) or share the nest with host young (low virulence). High-virulence brood parasite embryos exhibited higher overall metabolic rates than both non-parasitic (parental) species and low-virulence parasites. This was driven by significantly elevated metabolic rates around the halfway point of incubation. Additionally, a fine-scale analysis of the embryos of a host–parasitic pair showed faster increases in metabolic rates in the parasite. Together these results suggest that the metabolic patterns of the embryos of high-virulence parasites facilitate their early-life demands.

## 1. Introduction

The young of some species must hatch from their eggs with the physiological capacity to perform strenuous activities. Brood parasites are one such group where hatchlings may benefit from increased strength [1]. Brood parasitism occurs in birds, fishes, and insects, where parental care is costly and can be stolen [1–4]. In avian obligate brood parasitism, the parasitic progeny develop in the nest of their respective host species, exhibiting numerous adaptations to elicit sufficient parental care from their hosts [5]. Many brood-parasitic chicks face immediate demands on their physiology upon hatching, for which underdevelopment or any delay in growth would likely reduce chick viability. For instance, the parasitic chick’s survival is often dependent on reducing the feeding competition it faces from host young, such as by evicting them from the nest or killing them *in situ* [6,7]. For example, the nestlings of parasitic common cuckoos (*Cuculus canorus*) that are raised alongside host chicks have less chance of fledging than those that successfully eject nestmates [8,9]. Moreover, even those parasitic chicks that do not kill host chicks, such as cowbirds (*Molothrus* spp.), may need to out-compete their nestmates [10], which is also likely to be energetically demanding [11]. These energetic demands are likely higher than for non-parasitic altricial species, where competition among nestmates is lower.

Several aspects of embryonic physiology at the pre-hatching stage may enable parasitic chicks to cope with such early-life challenges. For instance, most parasitic embryos develop faster (and thus can hatch up to 30 h earlier and/or in a more developed state) than embryos of their hosts [12,13], perhaps due to having larger yolks as energy stores [14,15] and internal incubation of the egg prior to laying [12]. Additionally, lower parasitic eggshell conductance may conserve energy stores [16–19], and a greater frequency of embryonic movement potentially acts as a mechanism to increase a brood parasite embryo’s muscle development in preparation for killing or out-competing host offspring upon hatching [20]. However, it is currently unknown how changes in embryonic metabolic rate may facilitate such early hatching and muscle development.

The metabolic rates of developing embryos vary between species (e.g. [21]) and are linked to traits such as extended incubation periods due to long recesses between incubation bouts (e.g. Procellariiformes) [22], or whether a species is altricial or precocial [23]. The embryonic metabolic rate of altricial species generally increases continuously throughout incubation, whereas in precocial species metabolic rate increases very steeply for the first 75−80% of incubation, and then plateaus for the final 20−25% [24]. This elevated metabolic rate in precocial species is thought to support rapid development and strengthened musculature upon hatching [25]. As metabolic rate is directly translatable to the speed of development [21], elevated metabolic rates may be expected in all brood-parasitic species due to the requirements for early hatching, and the potential energetic demands placed upon the chick after hatching, whether these demands arise from evicting/killing host young, or out-competing them [26]. Alternatively, elevated metabolic rates may only be found in high-virulence parasites (defined as those which evict and/or kill the host young) due to the likely extreme energetic requirements of such behaviours, and the necessary muscle and bone development to undertake them. We therefore measured embryonic metabolic rates in a range of brood-parasitic species and their respective hosts, to test these two competing hypotheses.

## 2. Methods

### (a) Study species

We recorded metabolic rates for the embryos of 12 altricial bird species, of which five are obligate parasites and seven are hosts (parental/non-parasites) (table 1). Parasites were from four different families representing four of the seven independent evolutionary origins of avian brood parasitism [27,28]. Parasitic virulence was defined as ‘high’ if parasitic chicks typically kill host offspring (cuckoos, honeyguides), and ‘low’ if host offspring are typically raised alongside parasitic chicks (cowbirds, whydahs; table 1). Fieldwork was conducted in Zambia, the Czech Republic (Czechia) and the United States of America (USA) (table 1; see electronic supplementary material for full details).

**Table 1.** List of species, number of eggs for which at least one metabolic rate measurement was taken, parasitic and virulence status (assigned based on [26]), mean incubation length and country where measurements were taken. Host–parasite systems are indicated by matching letters.

species	no. of eggs	parasitic and virulence status	mean incubation length (days)	location	egg mass (g)
common waxbill (*Estrilda astrild*)	21	non-parasitic **^A^**	11.5	Zambia	0.68 ± 0.1
prothonotary warbler (*Protonotaria citrea*)	23	non-parasitic **^B^**	13	USA	1.9 ± 0.3
great reed warbler (*Acrocephalus arundinaceus*)	38	non-parasitic **^C^**	13	Czechia	2.85 ± 0.3
Eurasian reed warbler (*Acrocephalus scirpaceus*)	17	non-parasitic **^C^**	12	Czechia	1.69 ± 0.3
Zitting cisticola (*Cisticola juncidis*)	22	non-parasitic **^D^**	10	Zambia	0.91 ± 0.1
little bee-eater (*Merops pusillus*)	8	non-parasitic **^E^**	19	Zambia	2.1 ± 0.5
black-collared barbet (*Lybius torquatus*)	8	non-parasitic **^F^**	18	Zambia	3.66 ± 0.8
pin-tailed whydah (*Vidua macroura*)	15	parasitic, low virulence **^A^**	10	Zambia	0.97 ± 0.2
brown-headed cowbird (*Molothrus ater*)	23	parasitic, low virulence **^B^**	11	USA	2.61 ± 0.4
common cuckoo (*Cuculus canorus*)	53	parasitic, high virulence **^C^**	12	Czechia	3.16 ± 0.2
greater honeyguide (*Indicator indicator*)	6	parasitic, high virulence **^E^**	16	Zambia	3.45 ± 0.9
lesser honeyguide (*Indicator minor*)	12	parasitic, high virulence **^F^**	12	Zambia	2.9 ± 0.5

### (b) Respirometry

Metabolic rate was recorded repeatedly from the same eggs over the course of incubation (see electronic supplementary material, information for full details on timings and duration of metabolic rate measurements). Eggs were measured 1−6 times depending on egg survival and incubation length, and this was accounted for in statistical analyses (see below).

Embryonic metabolic rate was measured as CO_2_ production per minute (*V*CO_2_ ml min^−1^) using a portable flow-through (pull set up) respirometry system (‘FoxBox’, Sable Systems, USA) connected to a laptop computer [29–31]. The focal egg was placed into a 50 ml respirometry chamber through which air was pulled at a flow rate of 200−400 ml min^−1^ by the inbuilt pump of the FoxBox system. The airflow settings varied with the incubation stage and size of the eggs and this rate was corrected for in calculations of CO_2._ The excurrent air from the chamber subsequently passed through the respirometer where accurate flow rates and CO_2_ were measured at constant pressure, at a rate of one measurement per second. Full respirometry protocols and equipment details can be found in the electronic supplementary material.

### (c) Statistical methods

Data analysis was performed in R statistical software [32] using ‘R Studio’ [33]. Mean *V*CO_2_ over the most stable 4 min of the 12 min of recording was taken as the metabolic rate of the embryo at that time point. *V*CO_2_ was recorded as millilitre of CO_2_ per minute. To standardize across species with different incubation lengths, we scored embryonic development from 1 to 5 (electronic supplementary material, figure S1, electronic supplementary material, table S1) (following [7,20]). The area under the metabolic development curve was calculated following [34].

Phylogenetically controlled mixed models were used for the primary analysis to control for the non-independence of species. For this analysis, a phylogenetic tree of our focal species was constructed and downloaded from the online Tree of Life database, using the R package ‘rotl’ [35] (electronic supplementary material, figure S2). The phylogenetic signal of *V*CO_2_ was calculated as the proportion of the total variance in the trait that is explained by phylogeny (H^2^). This value is directly equivalent to Pagel’s *λ* [36,37]. The package ‘phyr’ was used to fit a phylogenetic mixed model (PMM), which accounts for intraspecific variation by permitting multiple measures per species to be included [36,37]. Log_10_-transformed mean *V*CO_2_ was the response variable in this model, with parasitic status (three levels: non-parasitic, low-virulence parasitic, high-virulence parasitic) and embryo stage (five levels; 1−5) as categorical predictor variables, and log_10_-transformed egg mass as a continuous covariate. The model also included random effects for phylogeny, species identity and ‘egg ID’ (as multiple measurements were taken for some eggs, see above). Embryo stage was included as a categorical rather than an ordinal variable to account for non-monotonic effects of embryo stage on metabolic rate (e.g. [37,38]). This model identified significant stage-by-status interactions (electronic supplementary material, table S2). These interactions were further explored using stage-specific PMMs that tested for effects of status on log_10_-transformed mean *V*CO_2_, with log_10_-transformed egg mass as a continuous covariate, and phylogeny, species identity and ‘egg ID’ as random effects. The difference in metabolic rate between the three groups (non-parasitic, low-virulence parasitic, high-virulence parasitic) was determined by comparing the change in model fit (assessed using the small-sample version of Akaike’s information criterion, AICc) [39].

An additional fine-scale analysis was performed to compare daily metabolic rate measurements taken from the eggs of common cuckoos and one of its hosts, great reed warblers (*Acrocephalus arundinaceus*). This finer-scale analysis was conducted on only this system because (i) daily measurements were possible and available, (ii) both host and parasite eggs were from the same nest, and (iii) all measurements were taken at equivalent developmental time points. A linear mixed model was used in this analysis, with ‘egg ID’ as a random effect to account for repeat measurements per egg. Incubation day (as a third-order polynomial) was applied as a continuous predictor (from day 2 to 14 from the start of incubation, defined as when the last egg in the clutch was laid). The difference in metabolic rate between species was determined by comparing the change in model fit (assessed using AICc [40]) associated with the introduction of interaction terms between species and each of the linear, quadratic and cubic terms in the model. Prior to analysis, data for mean *V*CO_2_ were adjusted for the effect of egg mass identified in the phylogenetic mixed model (note that these analyses were not corrected for multiple comparisons, following [41]).

## 3. Results

### (a) Comparison of embryonic metabolic rate between (discrete) incubation stages

**Figure 1.**
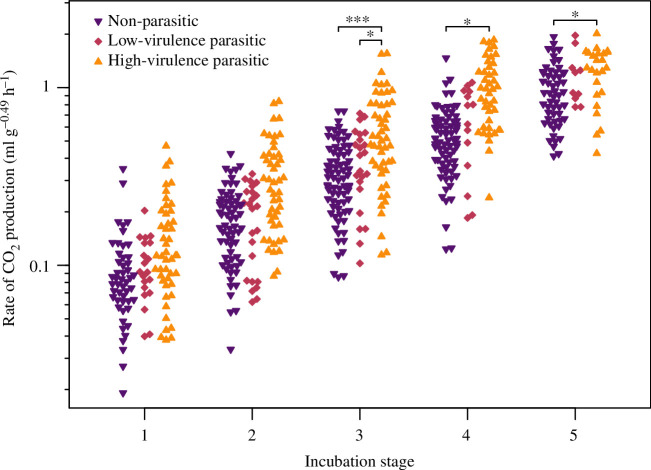
Metabolic rates at discrete incubation stages (see Methods) of eggs of non-parasitic, low-virulence parasitic and high-virulence parasitic species. High-virulence parasitic species differed significantly from both other groups at stage 3, and differed from non-parasitic species at stages 4 and 5 (* indicates *p* < 0.05; *** indicates *p* < 0.001). Raw data points are shown.

The embryos of high-virulence brood-parasitic species had greater metabolic rates than low-virulence brood parasites at incubation stage 3, and greater metabolic rates than non-parasites at incubation stages 3, 4 and 5 (figure 1; electronic supplementary material, table S2). There was no significant difference in metabolic rate between low-virulence brood parasites and non-parasites at any incubation stage (figure 1). There was negligible phylogenetic signal in embryonic metabolic rate (*H* ^2^ = 1.3 × 10^−5^; electronic supplementary material, table S2). The respective areas under the curve were 300.97 total CO_2_ production (ml g^–0.49^ h^–1^) for non-parasitic species, 285.86 total CO_2_ production (ml g^–0.49^ h^–1^) for low-virulence parasitic species and 405.31 total CO_2_ production (ml g^–0.49^ h^–1^) for high-virulence parasitic species, confirming that high-virulence parasites have higher energy budgets and energy consumption over the incubation period (30% and 34% higher than non-parasitic and low-virulence species, respectively). The increase in embryonic metabolic rate in high-virulence parasitic species appeared to level off at stage 4, with a plateau from stage 4 to stage 5 (figure 1). This pattern was not present for the embryos of non-parasitic and low-virulence parasitic species (figure 1).

### (b) Comparison of metabolic rate between common cuckoo and great reed warbler embryos

**Figure 2.**
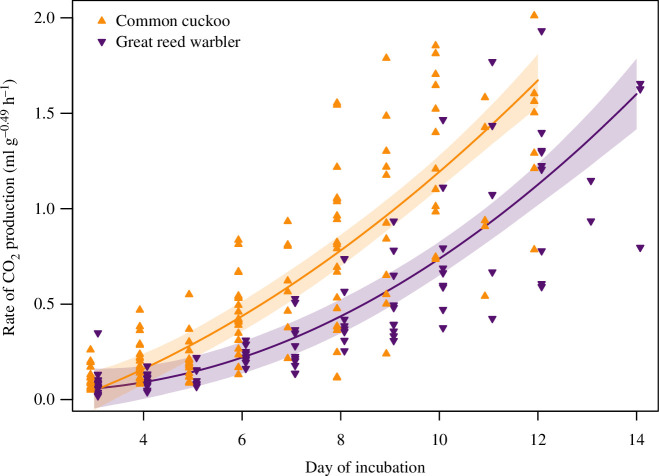
Mean metabolic rate over time (second-order polynomial of day into incubation) of common cuckoo (purple, downward-facing triangles) and great reed warbler (orange, upward-facing triangles) embryos. The embryonic metabolic rate of common cuckoos increased more steeply than that of their host, great reed warblers. Raw data points are shown. Shaded areas represent 95% bootstrap confidence intervals estimated from 10 000 simulations.

The metabolic rate of great reed warbler and common cuckoo embryos increased significantly throughout incubation in a non-linear fashion, and the rate of increase was faster for common cuckoos (figure 2). The best model was a quadratic model with species-specific linear and quadratic terms (AICc = 61), compared to a quadratic model with no species-specific terms (ΔAICc = 25.3), a cubic model with species-specific linear, quadratic and cubic terms (ΔAICc = 18.5) and a cubic model with no species-specific terms (ΔAICc = 28.3).

## 4. Discussion

High-virulence species had significantly higher metabolic rates late in incubation (stages 3, 4, 5) than non-parasitic species, as well as low-virulence species at stage 3. Overall, these findings do not support our first hypothesis that all brood parasites must develop faster to gain a competitive advantage against host chicks. Instead, they provide partial support for our second hypothesis that high-virulence brood-parasitic species need well-developed musculature and aerobic capacity to eliminate host offspring (e.g. [10,18,42]).

The differences in the ontogeny of metabolic rate between high- and low-virulence parasitic species at stage 3 of incubation suggest there are developmental differences amongst brood parasites which are likely linked to early post-hatch life demands. Little is known about the energetic costs a highly virulent parasite chick suffers through killing and evicting host offspring, nor about the costs of competitive begging by low-virulence parasites [10]. However, behavioural observations and growth measurements imply obvious strain for the chicks of high-virulence parasites. Video recordings of nestling greater honeyguides (*Indicator indicator*) killing the chicks of little bee-eaters (*Merops pusillus*) show the parasitic young breathing heavily and taking frequent rests [43]. Similarly, common cuckoo chicks, which often take several days to evict all the host offspring from the nest, suffer a reduced growth rate during this period [6,44,45]. Together, these observations suggest a high level of aerobic capacity is likely necessary to eliminate host young without incurring irreversible and long-term costs [44–47].

High levels of aerobic capacity upon hatching are also required by precocial species [47]. In accordance with this, the pattern of metabolic rate observed in high-virulence parasites (i.e. a steep increase for approx. 80% of the incubation duration, followed by a plateau) is similar to the pattern observed in precocial species [24,47]. The chicks of precocial species typically reach their approximate yolk-free hatching mass at this 80% time point [24]. Prior work has suggested that maturation of function in the sensory, neuromuscular and thermoregulatory systems of precocial embryos may require some time after tissue growth is essentially complete (i.e. yolk-free hatching mass is reached), but require relatively little energy [24,47]. This may account for the decline in growth rate and consequent decline or stabilization in metabolic rate before hatching [24,47], as seen in the high-virulence parasitic species. If high-virulence parasites do obtain approximate yolk-free hatching mass at incubation stage 4, this may permit high-virulence parasites to develop greater functional musculature prior to hatching, and/or have a more advanced cardiovascular system upon hatching. In precocial species, attaining yolk-free hatching mass earlier is thought to solidify muscle and bone development, to help with immediate movement and supporting body mass [24,47]. This may enable the chicks of high-virulence brood-parasitic species to cope with the energetic demands of ejecting or killing the host’s offspring. Such rapid development and muscular development may be facilitated in brood-parasitic embryos by greater energy reserves in freshly laid eggs [14]. Therefore, we might expect similar egg contents in high-virulence parasites and precocial species. However, the eggs of common cuckoos do not contain similar egg contents (e.g. yolk constituent components) to precocial species [14]. Therefore, it is still unclear how high-virulence brood parasites achieve their high metabolic rates.

Our study included measurements from multiple families of host and parasitic birds, and we accounted for their phylogenetic relationships statistically. In doing so, we found that phylogenetic signal in the pattern of embryonic metabolic rate among these species was negligible. The non-parasitic (host) species were, in most cases, phylogenetically distant from their parasite. It would, therefore, be useful for future research to measure metabolic rates in non-parasitic close relatives of high-virulence parasites, to determine whether the metabolic patterns observed are unique to parasitic species within these families, or whether an ancestral metabolic pattern predisposes them to evolving brood parasitism. Our discovery of a different pattern of metabolic rate development among high-virulence parasites also invites many new questions about how the physiology of these species differs from other birds, both as embryos and hatchlings. Future studies should investigate the physical and energetic demands of virulence behaviour both pre- and post-hatching to understand the selection pressures shaping the physiological differences. Such studies could include investigating the thermal biology of brood parasites, and establishing whether their chicks can exhibit the transitory shivering response (as precocial species do).

Beyond its importance for the evolution of brood-parasitic life history strategies, determining why the metabolic rate of high-virulence parasitic embryos differs from other species would enlarge our broader understanding of avian developmental trajectories (e.g. [48–51]). For example, determining whether there are shared physiological adaptations between precocial species and non-parasitic altricial species which experience high physical demands upon hatching (such as species where there is evidence of early jostling for begging position within a nest, siblicide or intense competition due to hatching asynchrony (e.g. [52]), could allow us to further establish whether and why similar developmental trajectories can convergently evolve across even distantly-related taxa under different selection pressures.

## Data Availability

All data are available in the electronic supplementary material [53].
